# Dermatan sulfate is a player in the transglutaminase 2 interaction network

**DOI:** 10.1371/journal.pone.0172263

**Published:** 2017-02-15

**Authors:** Grzegorz Wisowski, Ewa M. Koźma, Tomasz Bielecki, Adam Pudełko, Krystyna Olczyk

**Affiliations:** 1 Department of Clinical Chemistry and Laboratory Diagnostics, School of Pharmacy with the Division of Laboratory Medicine in Sosnowiec, Medical University of Silesia, Katowice, Poland; 2 Department and Clinic of Orthopedic and Trauma Surgery, School of Medicine with the Division of Dentistry in Zabrze, Medical University of Silesia, Katowice, Poland; University of Texas MD Anderson Cancer Center, UNITED STATES

## Abstract

Transglutaminase 2 (TG2) is a multifunctional protein that is primarily engaged in cell adhesion/signaling or shows Ca^2+^-dependent transglutaminase activity in the extracellular space of tissues. This latter action leads to the cross-linking of the extracellular matrix (ECM) proteins. The enhanced extracellular expression of TG2 is associated with processes such as wound healing, fibrosis or vascular remodeling that are also characterized by a high deposition of dermatan sulfate (DS) proteoglycans in the ECM. However, it is unknown whether DS may bind to TG2 or affect its function. Using the plasmon surface resonance method, we showed that DS chains, especially those of biglycan, are good binding partners for TG2. The interaction has some requirements as to the DS structure. The competitive effect of heparin on DS binding to TG2 suggests that both glycosaminoglycans occupy the same binding site(s) on the protein molecule. An occurrence of the DS-TG2 interaction was confirmed by the co-immunoprecipitation of this protein with native decorin that is a DS-bearing proteoglycan rather than with the decorin core protein. Moreover, *in vivo* DS is responsible for both TG2 binding and the regulation of the location of this protein in the ECM as can be suggested from an increased extraction of TG2 from the human fascia only when an enzymatic degradation of the tissue DS was conducted in the presence of the anti-collagen type I antiserum. In addition, DS with a low affinity for TG2 exerted an inhibitory effect on the protein transamidating activity most probably via the control of the accessibility of a substrate. Our data show that DS can affect several aspects of TG2 biology in both physiological and pathological conditions.

## Introduction

Transglutaminase 2 (TG2) is a multifunctional protein that is widespread in human tissues. Despite the lack of a leader sequence (secretion signal) in its polypeptide chain [1], TG2 is not only localized intracellularly but also exists in the extracellular space where it is associated with both the cell surface and the extracellular matrix (for review [2, 3]). An as yet unidentified TG2 translocation mechanism may involve recycling endosomes [4] but also depends on the N-terminal β sandwich domain of the protein [5], cysteine (Cys^277^) residue in the TG2 active site [6], the NO level [7] and/or the redox state [8]. Moreover, there is a positive correlation between the level of the TG2 expression and the proportion of the protein extracellular pool [9]. When externalized, TG2 plays two major roles–a Ca^+2^-dependent enzyme with a deamidating/transamidating activity and an enzymatically inactive signaling/adhesion molecule. The switch-over between these functions is accomplished through alterations in the TG2 molecule conformation from an extended (the so-called “open”) form with an exposed active center that comprises three residues Cys^277^, His^335^ and Asp^358^ to an enzymatically inactive compact (“closed”) form [10, 11]. The functional changes of TG2 in the extracellular space are also regulated by both Ca^+2^ and NO levels as well as by the redox status [12, 13]. A decreased Ca^+2^ concentration as well the NO-dependent S-nitrosylation or oxidation of some cysteine residues in TG2 molecules cause the inhibition of its transamidating activity. It seems that this activity is induced only transiently–the enzyme is usually in a latent state [14].

The extracellularly localized transamidase TG2 catalyzes the formation of the isopeptide links between a specific γ-glutamyl containing peptide substrate and an ε-amine group from a peptide-bound lysine or a free primary amine thus leading to the modification or cross-linking of many ubiquitous extracellular matrix (ECM) components such as fibronectin, fibrillar collagens or nidogen [8]. These structural modifications strongly affect the biomechanical properties of the ECM components and their susceptibility to proteolysis. Therefore, proper TG2 transamidating activity allows a stable tissue support to be formed that promotes cell adhesion, migration, growth and survival, and is crucial for correct wound repair or morphogenesis. In contrast, excessive TG2-dependent cross-linking can lead to arterial remodeling that is manifested by an increased stiffening of the arterial wall and a decreased arterial lumen diameter [8, 15] as well as to disturbances in the degradation and enormous accumulation of the ECM components, which results in tissue fibrosis [16, 17]. In addition, TG2 transamidating activity is involved in the stimulation of the activity of a major pro-fibrotic factor–transforming growth factor β (TGF β). This effect is caused by both the promotion of TGFβ expression via the TG2-dependent triggering of the NFκB pathway [17] and by an enhancing of the latent TGFβ activation [18]. Apart from its transamidation activity, TG2 also has an important non-enzymatic adapter/scaffolding function in the ECM. This reflects the formation of ternary complexes including TG2 that bind to the β1 or β3 integrin subunits and fibronectin [9] or to fibronectin and syndecan-4 [19]. These complexes are not only engaged in cell adhesion and intracellular signaling but also in the induction of the assembly of the fibronectin matrix [19,20]. Moreover, the complexes of the matrix deposited TG2 (and fibronectin) with the cell surface-localized syndecan-4 can compensate for the RGD peptide-induced loss of cell adhesion during the ECM remodeling that is associated with wound repair, angiogenesis or cancer metastasis, thereby rescuing cells from anoikis (cell death due to the absence of adhesion) [21]. In these complexes, TG2 directly binds to the heparan sulfate (HS) side chains of syndecan-4 and the binding is characterized by high affinity [22]. The interaction with the glycosaminoglycan chains of HS is also responsible to a great degree for the TG2 retention at the cell surface [22, 23]. Moreover, shedding of the HS chains as a result of the syndecan-4 core protein cleavage that is mediated by some matrix metalloproteinases is a mechanism by which TG2 is translocated into the ECM [23].

Dermatan sulfate (DS) like HS is a glycosaminoglycan (GAG) with a high content of the sulfate groups, which determines its high binding potential. However, it should be kept in mind that the mechanisms underlying the biological effects that are promoted by these GAGs can differ significantly. DS belongs to galactosaminoglycans, a subgroup of the GAG family, which also includes chondroitin sulfate (CS). DS is a copolymer of two types of disaccharide units–one containing N-acetyl galactosamine (GalNAc) and glucuronate (GlcA) residues and the other consisting of GalNAc and iduronate (IdoA) residues [24, 25]. In contrast, CS is only composed of GalNAc residues, which alternate with GlcA residues [24, 25]. DS chains and less often CS chains are covalently attached to the core proteins of two widespread matrix proteoglycans–decorin and biglycan. Via their DS/CS portion, decorin and biglycan influence the structure and properties of the ECM as well as cell behavior (for review see [24, 25]). Interestingly, DS/CS accumulation in the ECM occurs in processes such as wound healing or fibrosis [26], which are also accompanied by a high level of TG2 activity in the ECM [27]. However, it is unknown whether DS (and CS) can interact with TG2 and/or modulate its transamidating activity. This prompted us to investigate this issue.

## Materials and methods

### Preparation of human DS/CS

Samples of human fibrosis-affected palmar fascia were obtained from patients treated operatively for Dupuytren fibromatosis. Samples of normal fascia lata were collected from healthy individuals during reconstruction surgery following accidental injury. The experiments were undertaken with the understanding and written consent of each subject. The study methodologies conformed to the principles of the Declaration of Helsinki, and were approved by the Regional Ethical Committee of Medical University of Silesia. DSs from human fibrotic fascia decorin and biglycan were isolated as described previously [28]. Briefly: small DSPGs were extracted from combined tissue samples with buffered 7.8 M urea solution containing protease inhibitors and then fractionated firstly by an anion exchange chromatography on DEAE-Sephacel, subsequently by a gel filtration on Sepharose CL-4B and finally by a hydrophobic interaction chromatography on octyl Sepharose CL-4B. To release GAG chains the PG core proteins were digested with papain as described below. To some experiments the total DS/CS from the human fibrosis-affected palmar fascia were used. These GAGs were released from their core proteins by an exhaustive treatment of homogenized tissue material with papain in 0.1 M acetate buffer pH 5.5, containing 5 mM EDTA and 5 mM cysteine HCl. Proteolysis was conducted 2 × 24 h at 65°C and agitation. Peptides generated by papain as well as proteins resistant to the enzyme were precipitated with 7% (w/v) TCA and discarded after centrifugation. Then, GAGs and peptido-GAGs were precipitated with three volumes of ethanol. The obtained GAG pellets were exhaustively rinsed with 80% ethanol to remove TCA. To separate DS/CS from HS and hyaluronan the fascia GAGs were first submitted to combined action of heparinases I, II and III, and then treated with hyaluronidase from Streptomyces griseus, respectively. HS depolymerization was performed in 0.02 M Tris HCl buffer pH 7.5, containing 4 mM CaCl_2_ and 0.1% bovine serum albumin, for 6 h at 25°C. In turn, HA degradation was conducted in 0.05 M acetate buffer pH 6.0, containing 10 mM CaCl_2_, for 1 h at 37°C. All of the used enzymes were denatured by boiling for 3 minutes. Then, remaining fascia CS/DS were subjected to an anion exchange chromatography on DEAE-Sephacel column equilibrated in 0.05 M acetate buffer pH 6.0, containing 0.25 M NaCl, and eluted in 1.5 M NaCl. Fractions containing GAG were dialyzed and lyophilized. The obtained DS/CS were quantified by dimethylene blue reaction [29]. The purity of both the DS/CS preparation and all of the used standard GAGs was verified by electrophoresis on cellulose acetate in 0.034 M Al_2_(SO_4_)_3_ after specific enzymatic degradation.

### Analysis of porcine DS sulfation pattern

Commercial preparation of DS from porcine intestinal mucosa (Sigma, Germany) was firstly depolymerized by chondroitinase ABC in 0.05 M Tris HCl buffer, pH 8.0. Obtained DS disaccharides were then labeled with fluorophore 2-aminoacridone [30] and subjected to a reverse phase high performance liquid chromatography (RP HPLC) on PLRP-S 300 A column (4,6 mm×150mm; Polymer Laboratories, Varian, Shropshire, UK) equilibrated in 0.1 M ammonium acetate, run on a Varian ProStar HPLC system. After washing of the column with 2 ml gradient of 0–10% (v/v) methanol, disaccharides were eluted with 50 ml linear gradient of 10–30% (v/v) methanol and detected by in-line fluorescence (excitation at 425 nm, emission at 520 nm) [30].

### Analysis of DS/CS interactions with TG2 by the Surface Plasmon Resonance (SPR) method

Before a binding assay, the examined peptidoDS/CSs were biotinylated on their peptide portion. The reaction was conducted in the presence of 10 μl of 0.01M EZ-link Sulfo NHS-LC-Biotin (Thermo Scientific, USA) as a donor of active biotin, in 0.1 M PBS pH 7.2, for 2 h at a room temperature. Then, modified peptidoDS/CSs were precipitated with five volumes of ethanol. Biotinylation efficiency was estimated by EZ biotin quantitation kit (Thermo Scientific, USA). To examine their ability to bind to human recombinant TG2 (Zedira GmbH, Germany) the biotinylated peptidoDS/CSs were immobilized onto a streptavidin-coated sensor chip (SAP from Xantec, Germany) in the Springle Instrument (Autolab, Netherlands) and exposed to various concentrations of the protein. The binding assay was performed at a physiological ionic strength in PBS pH 7.4, containing 0.02% Tween-20 and 2 mM EDTA, at 21°C until equilibrium was reached. The dissociation phase of the TG2-DS/CS interaction was generated by a rapid replacement of the protein solution with the running buffer. The GAG surface was regenerated between particular measurements by washing with 2 M NaCl in the running buffer. Blank sensorgrams that were subtracted from DS/CS sensorgrams were obtained after an addition of the TG2 solutions onto sensor surface unmodified by GAGs. Kinetics parameters of the examined reactions (i.e. association and dissociation rate constants k_a_ and k_d_, respectively) were calculated from the association phase of the sensorgrams using the non-linear curve-fitting software supplied with the instrument. The equilibrium dissociation constant, K_D_ was obtained from the ratio of k_d_/k_a_. To estimate the influence of mass transport limitation on the binding experiments, the plots of relative response values as a function of the used TG2 concentration were constructed [31] for all of the examined DS/CSs. Linear dependence between these parameters observed for all of the examined GAGs clearly indicated that the binding is not limited by mass transport. To evaluate the competition of heparin for the interaction between the DS/CS and TG2 the total fibrotic fascia DS/CS was immobilized onto the sensor chip and exposed to 150 nM of TG2 which was premixed with increasing amounts of heparin (the average molecular weight 5 kDa).

### Solid-phase binding assay

The porcine DS was dissolved in PBS, pH 7.4 and aliquots (100 μl) containing 0.15 μg of this GAG were subsequently put into wells of a 96-well microplates (Immulon 2HB, Thermo Labsystems, USA) for coating. Plates were incubated overnight at 4°C and the coating efficiency was verified by a reaction of the adsorbed DS with dimethylmethylene blue [29]. Then the wells were extensively washed with PBS containing 0.05% Tween-20 (PBST) and 2 mM EDTA, and blocked for 1h with 1% BSA in PBST. Subsequently the plates were incubated for 2h at a room temperature with various concentrations of TG2 dissolved in PBST, containing 1% BSA. After incubation the wells were rinsed five times with PBST, and the bound TG2 was detected by a rabbit polyclonal anti-human TG2 antibody (Thermo Fisher, PA5-16272, USA) used at a concentration of 5 μg per 1 ml of PBST, containing 1% BSA, for 1h at room temperature. After an extensive washing (five times) with PBST the secondary antibody (a peroxidase-conjugated goat anti-rabbit IgG) at a dilution of 1:50 000 was added to the wells and the plates were incubated for 1h at a room temperature. The formed immunological complexes were detected by a colorimetric reaction with peroxidase substrate 3,3’,5,5’-tetramethylbenzidine (absorbance was measured at 450 nm). Nonspecific binding was estimated in BSA-coated wells, and was subtracted from absorbance values measured in the DS-coated wells. Moreover, TG2 binding in the BSA-coated wells represented the negative control for the DS-TG2 interaction whereas TG2 binding in the fibronectin-coated wells demonstrated its positive control.

### Co-immunoprecipitation of TG2 with decorin from culture medium

Human keloid fibroblasts (Kel Fib, ATCC CRL-1762, LGC Standards, Poland) were cultured in DMEM supplemented with 1% fetal bovine serum and 20 μM GM6001 (Tocris, USA) or alternatively 50 μM putrescine. After an addition of protease inhibitors and regulation of EDTA concentration to 4 mM, the conditioned culture medium was concentrated with a centrifugal concentrator Amicon Ultra-15 (Merck Millipore Ltd., Germany). Then quantification of GAGs by reaction with dimethylmethylene blue [29] was made and one of the two equal portions of the obtained medium concentrate (each containing 5 μg of GAGs) was treated with chondroitinase ABC (0.01 units per 1 ml of the conditioned medium initial volume) for 3 h, at 37°C, under agitation to destroy DS/CS chains. Both portions of the medium concentrate were subsequently submitted to immunoprecipitation with a mouse monoclonal anti-human decorin antibody (R&D Systems, MAB143, USA). However, in some experiments before the immunoprecipitation to both portions of the medium concentrate an exogenous human recombinant TG2 was added (1.5 μg) and the samples were incubated for 0.5 h at 25°C to form decorin-TG2 complexes. Moreover, before the immunoprecipitation all of the medium samples were pre-cleared with Protein G-coupled Sepharose. The immunoprecipitation was conducted overnight at 4°C, and immunological complexes were subsequently captured on Protein G-coupled Sepharose. Then, immunoprecipitates were separated by reducing SDS-PAGE on 4–15% gradient gel and probed with the antibody against human decorin or a rabbit polyclonal anti-human TG2 antibody (Thermo Fisher, PA5-16272, USA) after electrotransfer to PVDF membrane. The electrotransfer of immunoprecipitates and their Western blotting were carried out as described previously [26]. The horseradish peroxidase-conjugated secondary antibodies (a goat anti-mouse or goat anti-rabbit antibody) were used at dilution of 1:80 000. The formed immunological complexes were detected by a reaction with peroxidase substrate 3,3’,5,5’-tetramethylbenzidine (TMB; solution for membranes (Sigma, Germany)). Some blots were stripped and re-probed with another antibody followed by chemiluminescence.

### Extraction of TG2 from normal human fascia

The protein was extracted from a tissue samples treated with chondroitinase ABC or heparinase III to remove fascia DS/CS or HS, respectively. The extraction in the presence of the former enzyme (0.1 U/ml) was conducted in 0.05 M Tris HCl buffer pH 8.0, containing protease inhibitor cocktail, for 24 h at 37°C. In some experiments the extraction solution also contained a rabbit polyclonal anti-human fibronectin antibody (Santa Cruz Biotechnology, sc-9068) used at 15 μg/ml and/or a mouse monoclonal anti-human type I collagen antibody (Santa Cruz Biotechnology, sc-59773, USA) applied at 4 μg/ml. The release of TG2 from the tissue samples treated with heparinase III (0.5 U/ml) was carried out in 0.02 M Tris HCl buffer pH 7.5 with protease inhibitors for 24 h, at 25°C. Components extracted from the fascia samples were subsequently precipitated with five volumes of ethanol and analyzed by reducing SDS-PAGE and Western blotting using a rabbit polyclonal anti-human TG2 antibody (Thermo Scientific, PA5-16272, USA) at a concentration 5 μg/ml. In turn, the second antibody (a horseradish peroxidase (HRP)-conjugated goat anti-rabbit antibody) was applied at a dilution 1:80 000. The formed immunological complexes were detected by the reaction with TMB. The obtained blot were submitted to densitometric analysis.

### Analysis of DS/CS effect on TG2 transamidating activity using CBZ-Gln-Gly as amine acceptor

The impact of DS/CSs on the TG2-mediated incorporation of biotinylated cadaverine to CBZ-Gln-Gly as an amine acceptor was evaluated by the use of a commercial colorimetric microassay test (Covalab, France). In brief: to wells of a microtiter plate, which were covalently coupled with aforementioned amine acceptor, 0.05 mU of the human recombinant TG2 (Zedira GmbH, Germany) and different amounts of the porcine DS or the total DS/CS from the fibrosis-affected human fascia were applied. The transamidation reaction was conducted in the presence of CaCl_2_ and dithiothreitol for 15 min, at 37°C, and stopped by addition of EDTA. The level of the incorporated biotin-cadaverine was detected by the use of HRP-conjugated streptavidin followed by an incubation with substrate for the enzyme.

### Analysis of DS/CS impact on TG2-dependent transamidating activity toward collagen

In order to further evaluate the DS/CS-mediated influence on TG2 action, the incorporation of biotinylated cadaverine into collagen type I was tested in the presence or the absence of the GAGs. Briefly: wells of a microtiter plate were coated with 100 μl of 0.1% collagen solution at 4°C overnight. After an exhaustive rinsing with 0.05 M Tris HCl buffer, pH 7.5, containing 0.1% Tween 20, the wells were blocked with 3% BSA in the same buffer for 30 min. Then, to each well 100 μl of 0.05 M Tris HCl buffer pH 7.5, containing 0.1% Tween 20, 5 mM CaCl_2_, 10 mM DTT, 0.1 mM biotin-cadaverine, 0,04 mU of human recombinant TG2 (Zedira GmbH, Germany) and/or appropriate amount of GAGs was applied and the plate was incubated for 30 min at 37°C. The enzymatic reaction was stopped by washing twice with 250 μl of 50 mM EDTA and the wells were rinsed 3 times with 0.05 M Tris HCl buffer, pH 7.5, containing 0.1% Tween 20. Next, the streptavidin-HRP conjugates, which were diluted at 1:50 000 in 0.05 M Tris HCl buffer pH 7.5, containing 0.1% Tween 20 and 0.1% BSA, were added to each well and the plate was incubated by 1h at room temperature. Subsequently, the wells were washed 3 times with 0.05 M Tris HCl buffer, pH 7.5, containing 0.1% Tween-20, and 100 μl of HRP substrate solution (TMB) was added to each well. After incubation for 30 min at a room temperature the reaction was stopped by an addition of 100 μl of 1 M HCl. The amount of the incorporated cadaverine was quantified by absorbance measuring at 450 nm in a plate reader.

### Statistical analysis

The data were analyzed using the Shapiro-Wilk’s test to verify the assumption of normal distribution. The results were expressed as the mean values ± SD. Between-group comparisons were based on a one-way ANOVA and post-hoc the Tukey’s HSD test with p < 0.05 as significant.

## Results

### DS is a binding partner for TG2

**Fig 1:**
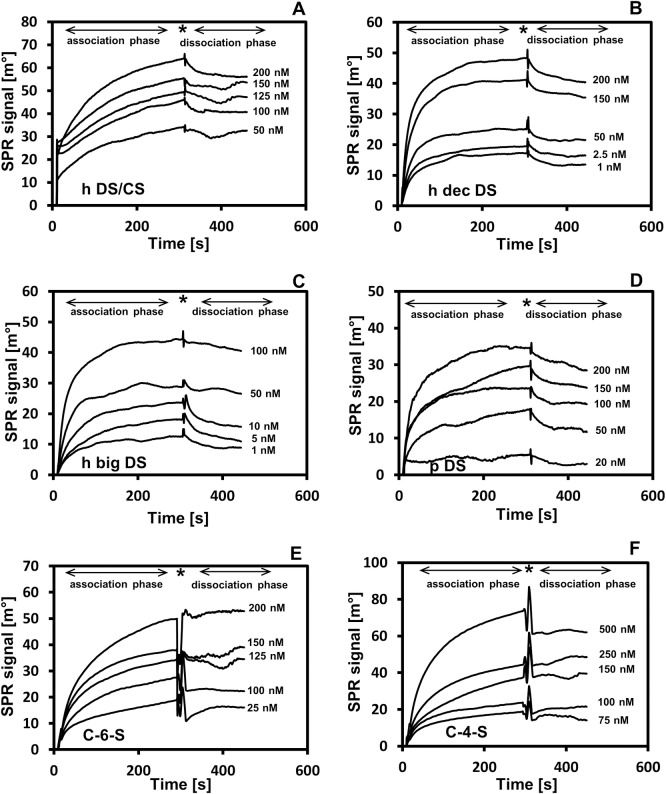
Surface plasmon resonance (SPR) analysis of interactions between various DS/CS and TG2. DS/CS chains that were biotinylated on their core protein remains were immobilized onto streptavidin-modified sensor chip and exposed to various concentrations of TG2 at a physiological ionic strength and 21°C, as described in Materials and methods. The dissociation phase of these interactions was generated by a rapid replacement of the TG2-containing solution with running buffer. (A) h CS/DS–sensorgrams obtained for the total human fibrotic fascia CS/DS; (B) h dec DS–sensorgrams obtained for human decorin DS from the fibrotic fascia; (C) h big DS–sensorgrams obtained for human biglycan DS from the fibrotic fascia; (D) p DS–sensorgrams obtained for porcine intestinal mucosa DS; (E) C-6-S–sensorgrams obtained for C-6-S from shark cartilage; (F) C-4-S–sensorgrams obtained for C-4-S from whale cartilage. Arrows show the association and dissociation phase of examined interactions. Asterisk shows signal reaction on a solution exchange.

**Fig 2:**
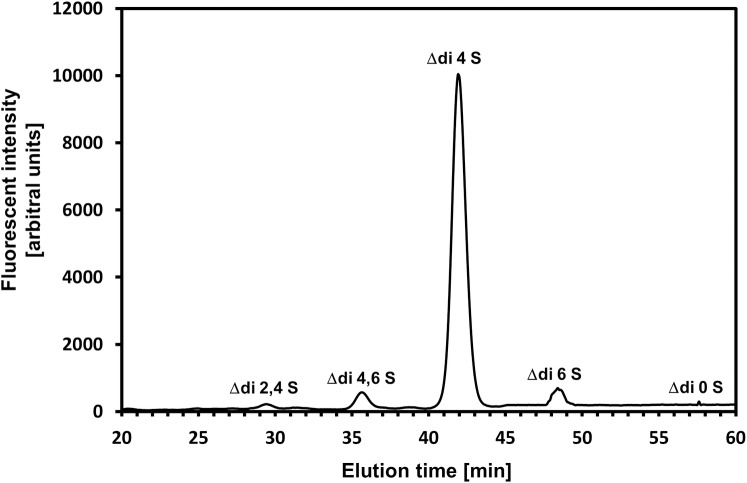
Typical reversed phase HPLC analysis of the porcine DS sulfation pattern. The GAG was extensively depolymerized with chondroitinase ABC, and the released unsaturated disaccharides were labeled with fluorophore 2-aminoacridone and separated by HPLC, as described in Materials and methods. The elution positions of individual disaccharides are indicated. The percentage content of individual disaccharides is as follow: Δdi2,4S – 1.4±0.2%; Δdi4,6S – 3.7±0.2%; Δdi4S – 88.4±0.1%; Δdi6S – 5.7±0.6%; Δdi0S 0.8±0.3% (mean ± S.D. of five independent analyses).

In order to investigate whether DS/CS binds to TG2, we chose the total DS/CS prepared from a Dupuytren disease-affected human palmar fascia. This disease, which represents palmar fibromatosis, is characterized by both a high accumulation of DS/CS in the ECM and an enhanced activity of TG2 [26, 27]. To examine the DS/CS-TG2 interaction, we used the surface plasmon resonance (SPR) method with the GAG immobilized onto a sensor chip and exposed to various concentrations of the enzyme under a physiological ionic strength. However, the SPR instrument that was used in our experiment has a cuvette-type design. Because of this special design, ligand re-binding interferes in the dissociation phase of the interaction being examined that leads to a reduction in the slopes of the dissociation phase curves. Thus, the kinetics parameters of the reaction were calculated based on the association phases of the sensorgrams that were obtained (Fig 1) according to the recommendation of the SPR instrument supplier. The data obtained (Table 1) clearly show that the total DS/CS from fibrosis-affected fascia is a good binding partner for TG2 as can be concluded from the low value of the equilibrium dissociation constant K_D_, which characterizes this interaction. Nevertheless, the interaction occurs at a relatively low association rate and equilibrium is slowly reached (Figs 1A and 4A). Since, the total fascia DS/CS applied was a mixture of side chains that were primarily derived from two small leucine-rich proteoglycans–decorin and biglycan [26], in the next step, we tested the binding potential of particular PG. To achieve this goal, we used an urea-extractable pool of each PG as the donor of DS chains that were to be applied in SPR experiment (Fig 1). The calculated kinetics parameters that characterized of the interactions examined (Table 1) showed that the DS chains from both the fibrotic fascia decorin and biglycan demonstrated high affinity for TG2, although the latter PG seems to be a slightly better binding partner for the enzyme. In addition, the K_D_ values that described the interactions of biglycan and decorin DSs with TG2 were lower than the value of K_D_ that characterized the total fascia DS/CS binding to the protein (Table 1). This observation suggests that the tissue pool of DS/CS includes subpopulations that may significantly differ in respect to their binding potential toward TG2. Therefore, to verify the hypothesis that special structural features of DS/CS chains are required in order to have the ability to bind TG2, we evaluated the binding potential that is associated with structurally different chondroitin-dermatan glycans using the SPR method. In addition to the fibrotic fascia decorin and biglycan GAGs, DS from porcine intestinal mucosa, C-6-S from shark cartilage and C-4-S from whale cartilage were also included in the examination. Our previous studies [32] found that all of the included GAGs differ significantly in regard to their glucuronosyl epimerization patterns. Both CSs are completely devoid of conformationally flexible IdoA residues. In turn, the porcine DS has the highest content of IdoA residues (92% of the total hexuronate residues), whereas the fascia decorin GAG is characterized by the lowest number (78%) of such constituents among all of the DSs applied [32]. Moreover, both human DSs have IdoA-containing disaccharides that are assembled into short chain sections, whereas the porcine DS demonstrates an equal proportion of both large and small “IdoA sections” [32]. In addition to the epimerization patterns, all of the GAGs examined differ substantially in respect to their sulfation patterns. The results of a RP HPLC analysis of the 2-aminoacridone tagged disaccharides that were generated by chondroitinase ABC (Fig 2) showed that the porcine DS is primarily composed of 4-O-sulfated disaccharides (~88% of the total disaccharides) (Fig 2). Moreover, this GAG is characterized by a high content of 4,6-O-disulfated disaccharides (Fig 2). Our previous study [33] showed that both human DSs contain a very high level of 4-O-sulfated disaccharides typically for such a kind of GAG (more than 85% of the total disaccharides). On the other hand, these DSs have a higher content of 2,4-O-disulfated disaccharides (7.6% and 6.5% in the case of decorin and biglycan GAG, respectively) but a lower level of 4,6-O-sulfated units (0.8% and 0.4% in the case of decorin and biglycan GAG, respectively) [33] as compared with the porcine GAG (Fig 2). Moreover, another study [28] allowed us to determine that in comparison with the other GAGs that were examined, the shark C-6-S has the highest content of both 6-O-sulfated and 2,6-O-disulfated disaccharides, which represent 48% and 14% of the total disaccharide units, respectively [28]. In addition, this GAG contains large quantities of 4-O-sulfated (28.7%) and 2,4-O-sulfated (4.92%) disaccharides [28]. In turn, the whale C-4-S has a large amount of 4-O- and 6-O-sulfated disaccharides (77 and 19%, respectively) [28]. In addition, this C-4-S is characterized by the lowest level of 2,4-O-disulfated disaccharides (0.11%) among all of the GAGs applied [28]. All of the DS/CSs that were examined also differed in their molecular weight as can be seen from the significant differences in electrophoretic mobility among them during PAGE (data not shown). Based on a comparison of the electrophoretic mobility of these GAGs with that characterizing the standard DS with a known molecular mass, we calculated the average Mr values, which are as follows: human decorin DS from fibrotic fascia– 18 kDa; human biglycan DS from fibrotic fascia– 15 kDa; the total DS/CS from fibrotic fascia– 16 kDa; porcine intestinal mucosa DS– 36 kDa; shark cartilage C-6-S– 42 kDa and whale cartilage C-4-S– 38 kDa. On the other hand, in the above experiment, we omitted the normal fascia DS/CS because this GAG is structurally “too similar” to that of the fibrotic tissue [28].

**Table 1:** Kinetic parameters characterizing the interactions of transglutaminase 2 (TG2) with structurally different types of dermatan sulfate (DS)/ chondroitin sulfate (CS).

DS/CS	Association rate constant k_a_ (M^-1^s^-1^)	Dissociation rate constant k_d_ (s^-1^)	Equilibrium dissociation constant K_D_ (nM)
The total DS/CS from fibrotic human fascia	3,6±1,5×10^5^	2,5±1,1×10^−2^	70,6±9
DS from fibrotic human fascia decorin	4,4±2×10^5^	2±1×10^−2^	41,5±18
DS from fibrotic human fascia biglycan	4,0±1.6×10^5^	3,4±1,2×10^−3^	11,1±6
Porcine intestinal mucosa DS	2,2±1,3×10^5^	2,8±1,4×10^−2^	142,6±30
Shark cartilage C-6-S	1,5±0,1×10^4^	0,7±0,2×10^−3^	452±109 *
Whale cartilage C-4-S	1,2±0,2×10^3^	9,8±1×10^−3^	8200±900 *

The interactions of the immobilized DS/CS with TG2 were determined through the surface plasmon resonance (SPR) method, as detailed in Materials and methods. The interaction parameters were calculated using the nonlinear curve fitting software supplied with the SPR instrument.

* differences statistically significant compared to all of the remaining GAGs (p ≤ 0.05).

The examination of the DS/CS binding to TG2 using the SPR method (Fig 1) revealed that the shark C-6-S have a markedly lower affinity for the protein compared to all of the tested DSs as can be seen from the substantially higher K_D_ values that characterized its interaction (Table 1). However, the highest K_D_ was found for the interaction between the whale C-4-S and TG2 (Table 1), thus indicating that this GAG has the lowest affinity for the protein among all of the examined GAGs. Thus, the data that were obtained strongly support the relevance of a special fine structure of the DS/CS chains in their interactions with TG2.

**Fig 3:**
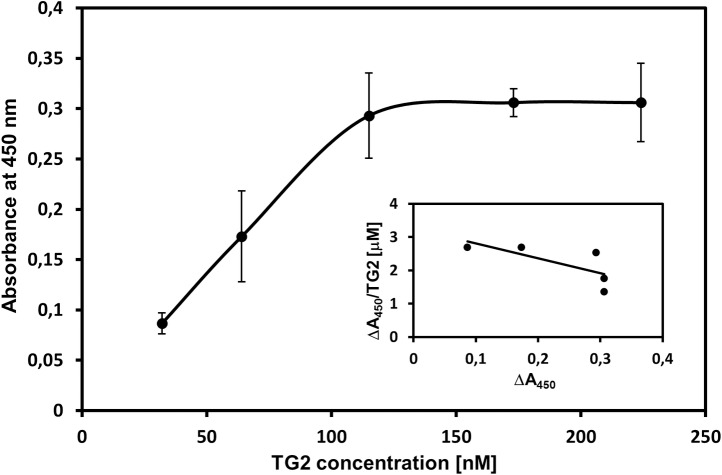
Typical saturation binding of TG2 to the immobilized DS. Incubation of the DS with an increasing amounts of TG2 was conducted, as described in Materials and methods. The analysis was carried out in the form of three independent experiments performed in duplicate. Data that are shown represent results (the mean ± S.D.) of one of them. The inset shows a Scatchard-type plot of the experimental data for the porcine DS binding to TG2.

To confirm the ability of DS to bind to TG2, we also tested this interaction using a solid-phase binding assay with this GAG non-covalently immobilized to wells in a plastic microplate. However, due to the fact that this method consumes a great amount of GAG material, we could only analyze the binding of the porcine DS since we only had a sufficient quantity of this glycan. Fig 3 presents the typical binding curve that describes the DS-TG2 interaction, which is TG2 concentration-dependent and saturable. The Scatchard-type plot was drawn based on this binding curve (Fig 3 inset) and the dissociation constant K_D_ for the porcine DS-TG2 interaction was calculated as was previously described [28]. The K_D_ value 155±57 nM that was obtained is in good agreement with this parameter as determined using the SPR method (Table 1).

**Fig 4:**
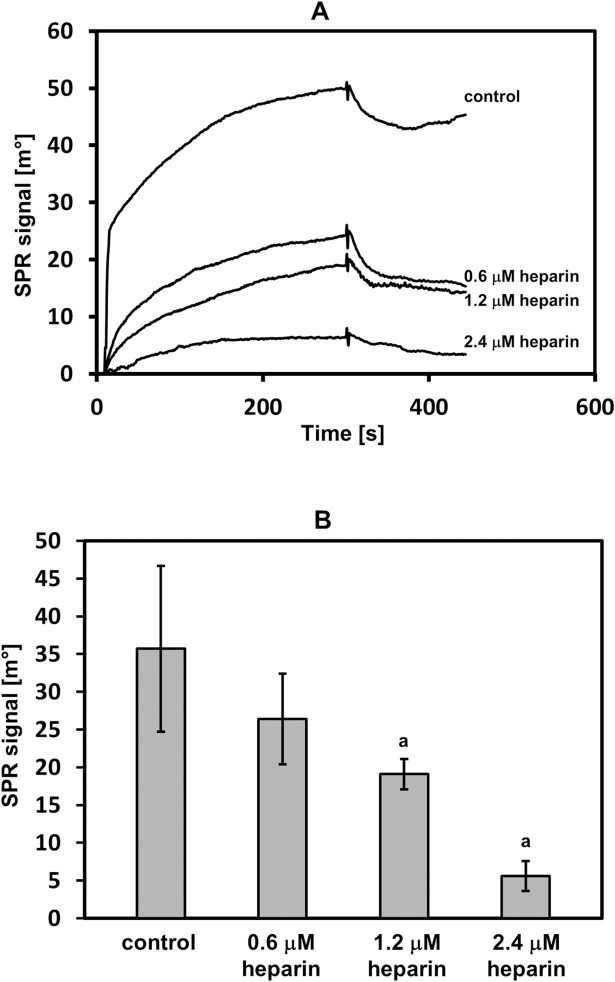
The competition of heparin for the interaction between the DS and TG2. The DS that was immobilized onto sensor disc was exposed to 150 nM of TG2 without heparin (control) or with indicated amounts of heparin, as described in Materials and methods. (A) Representative sensorgrams that show the interaction between the DS and TG2 in the presence of heparin. (B) The interference of heparin in the DS-TG2 interaction was estimated as reduction in the surface plasmon resonance (SPR) signal. The results (the observed SPR response) are means ± S.D. of at least 3 independent experiments. a–statistically significant differences compared to the binding without heparin (p ≤ 0.05).

Heparin, which is a strong binding partner for TG2 [22], most probably interacts with two distinct sites on the enzyme molecules [23,34]. The first binding site includes two short sequences and is formed when TG2 adopts a closed conformation [34] especially after GTP binding. The other heparin-binding site is localized in a single amino acid sequence [23]. Then, we tested whether DS can occupy the heparin-binding site(s) on TG2 molecules. To achieve this goal, we evaluated the competitive effect of heparin for the interaction between the immobilized fibrotic fascia DS and TG2, which was measured in the absence of GTP and DTT as a reduction in the SPR signal, which indicated a decrease in TG2 binding to DS. As can be seen in Fig 4, heparin was able to interfere with DS binding to TG2 at the majority of the concentrations that were applied. These data suggest that both GAGs, which are highly negatively charged molecules, can occupy the same cluster(s) of basic amino acids on the protein molecules.

**Fig 5:**
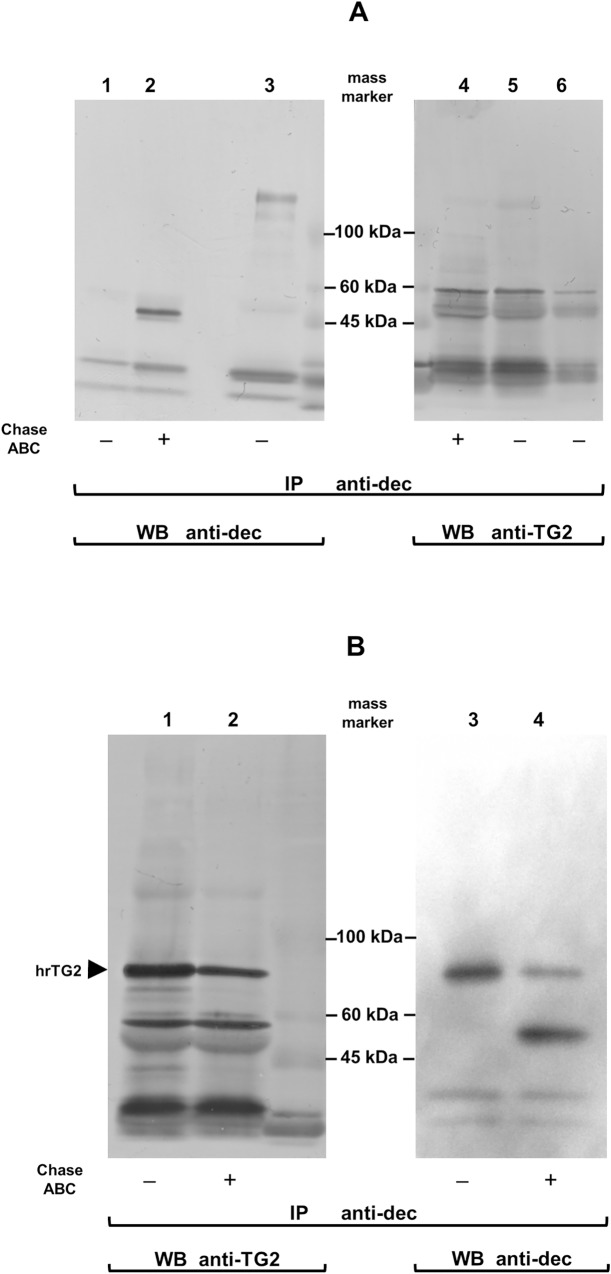
TG2 is associated to DS/CS side chains of decorin molecules secreted into the culture medium by human keloid fibroblasts. (A) Molecular complexes that contained native decorin or decorin treated with chondroitinase ABC (Chase ABC) to eliminate DS/CS chain were immunoprecipitated (IP) with an anti-human decorin core protein antibody (anti-dec) from the equal portions of the conditioned medium (each portion contained 5 μg of GAG) and separated by reducing SDS-PAGE followed by Western blot detection with the anti-dec or an anti-human TG2 serum. Lane 1 –the anti-dec antibody applied as negative control; lane 2 –the core protein of decorin; lane 3 –native decorin; lane 4 –components that were co-immunoprecipitated with the decorin core protein and detected by the anti-TG2 antibody; lane 5 –molecules that were co-immunoprecipitated with native decorin and detected by the anti-TG2 antibody; lane 6 –the cross-reactivity between the antibody that was used for the co-immunoprecipitation (mouse anti-decorin) and the sera were applied for the Western blotting (rabbit anti-human Tg2 and goat anti-rabbit IgG). The migration position of mass markers is shown. (B) The equal portions of the conditioned medium (each containing 5 μg of GAG) from the keloid fibroblasts, which have been cultured in the presence of 50 μM putrescine, were untreated or digested with Chase ABC and subsequently incubated with a human recombinant TG2, as described in Materials and methods. Molecular complexes that contained decorin or its core protein were immunoprecipitated and subjected to Western blot analysis firstly for TG2, and after stripping for decorin. Lane 1 –TG2 that co-immunoprecipitated with the untreated decorin; lane 2 –TG2 that co-immunoprecipitated with Chase ABC treated decorin; lane 3 –decorin untreated with Chase ABC; lane 4 –decorin digested with this enzyme. The migration position of the human recombinant TG2 (hrTG2) is indicated by arrowhead. All the experiments were performed in triplicate.

To corroborate the biological occurrence of a TG2 interaction with DS/CS, we studied the co-immunoprecipitation of the enzyme with decorin, which was secreted into the culture medium by keloid fibroblasts. Firstly, we tested the medium from the cells that had been cultured in the presence of GM6001, which is an inhibitor of several MMPs since these enzymes play significant roles in the degradation of TG2 [35]. Decorin was immunoprecipitated from the conditioned medium with an antibody against its core protein before and after the chondroitinase ABC-mediated elimination of the GAG moiety and then the precipitated molecules were resolved using SDS-PAGE followed by their electrotransfer into the PVDF membrane. The blots that were obtained were probed with anti-decorin or anti-TG2 serum. On the blots of immunoprecipitates that had not been treated with chondroitinase ABC (Fig 5A, lane 3), the anti-decorin antibody identified several species that had molecular masses from 81 to 148 kDa. The bands of these components were effectively eliminated by chondroitinase ABC, which yielded the appearance of a characteristic ~ 47 kDa decorin core protein doublet (Fig 5A, lane 2). In turn, on the blots of immunoprecipitates that had not been treated with chondroitinase ABC, the anti-TG2 antibody revealed the presence of a barely visible component (Fig 5A, lane 5) that showed electrophoretic mobility corresponding to that of the major decorin band at 148 kDa (Fig 5A, lane 5 versus lane 3). These results indicate that at least the majority if not all TG2 molecules that interacted with decorin in the keloid fibroblast culture medium were covalently coupled to the core protein of this PG, thus forming complexes that were resistant to the dissociation effect of the components that were present in the SDS-PAGE sample buffer such as the sodium dodecyl sulfate (SDS) and DTT. Moreover, this suggestion is further supported by our observation that the chondroitinase ABC-dependent elimination of DS prior to the decorin immunoprecipitation did not affect the ability of this PG to react with anti-TG2 serum (Fig 5A, lane 4). Therefore, the results that were obtained indicate that the decorin core protein is at least a substrate for TG2 transamidating activity if not the binding partner for this enzyme. However, these data do not exclude the possibility that decorin DS can be an additional or even the initial acceptor of TG2. In order to verify this hypothesis, we repeated the above experiment using the medium from the keloid fibroblasts that had been cultured in the presence of putrescine, which is a competitive amine inhibitor of TG2 transamidating activity. Moreover, in order to enhance the number of potential decorin-TG2 complexes, we added the human recombinant enzyme to the conditioned culture medium prior to the decorin immunoprecipitation. The data obtained (Fig 5B) clearly show that native decorin co-immunoprecipitated with TG2 more frequently than this PG that had been treated with chondroitinase ABC. However, as was expected, the major decorin fraction that was immunoprecipitated from the conditioned medium of the cells cultured in the presence of putrescine displayed a higher electrophoretic mobility than the one that characterized the major decorin fraction from the medium of the cells that had been grown without putrescine (Fig 5B, line 3 versus Fig 5A, line 3). The observed alteration in decorin mobility is most probably associated with the putrescine-dependent blockage of the TG2-mediated protein cross-linking to the decorin core protein.

**Fig 6:**
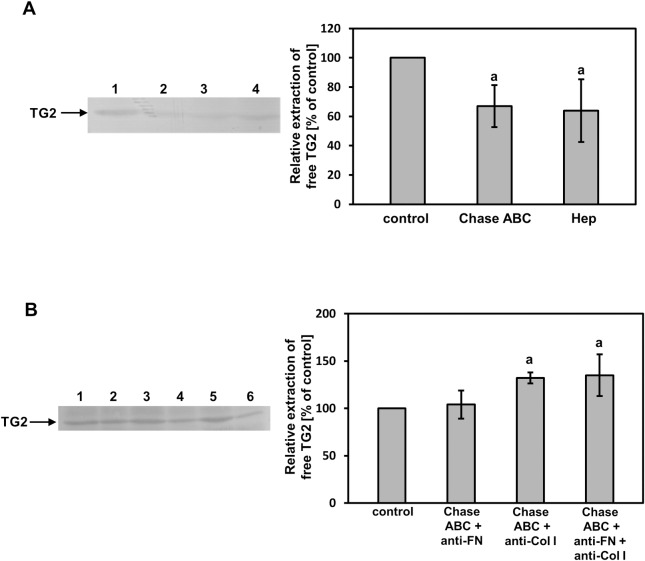
Acceptors of TG2 in the extracellular matrix of human fascia. (A) The protein was extracted from tissue samples that were treated with chondroitinase ABC (Chase ABC) or heparinase III (Hep) to eliminate DS/CS or HS, respectively. Components that were extracted from 1 mg of the dry tissue were subjected to reducing SDS-PAGE and Western blotting using the anti-human TG2 antibody, as described in Materials and methods. Lane 1 –an extraction with buffer, pH 8.0; lane 2 –an extraction with buffer, pH 8.0 and Chase ABC; lane 3 –an extraction with buffer, pH 7.5 and heparinase III; lane 4 –an extraction with buffer, pH 7.5. Arrow indicates the migration position of TG2. Relative quantification of the free TG2 extractability is presented as means ± S.D. of at least three independent experiments. a–differences statistically significant versus control (buffer treated tissue samples) (p ≤ 0.05). (B) TG2 that was extracted from 1 mg of the dry human fascia after Chase ABC-dependent degradation of tissue DS/CS in the presence of anti-fibronectin (anti-FN) and/or anti-collagen I (anti-Col I) antibodies was separated by reducing SDS-PAGE followed by Western blotting with the anti-human TG2 antibody. Lane 1 –an extraction with anti-fibronectin and Chase ABC; lane 2 –control extraction with anti- fibronectin; lane 3 –an extraction with anti-collagen I and Chase ABC; lane 4 –control extraction with anti-collagen I; lane 5 –an extraction with anti-fibronectin, anti-collagen I and Chase ABC; lane 6 –control extraction with anti-fibronectin and anti-collagen I. Arrow indicates the migration position of TG2. Relative quantification of the free TG2 extractability is presented as means ± S.D. of five independent experiments. a–differences statistically significant versus control (tissue samples treated only with proper antibody and buffer) (p ≤ 0.05).

To further examine the *in vivo* occurrence of DS/CS binding to TG2, we studied whether the chondroitinase ABC-dependent elimination of these GAGs affects TG2 extractability from tissue samples. For this experiment, we selected normal human fascia because of the expected lower cross-linking of the matrix components in this tissue than in fibrosis-affected fascia, which should yield a higher extractability of TG2. Unexpectedly, the enzymatic elimination of fascia DS/CS resulted in a reduction of TG2 extractability as compared with the control buffer extracted tissue samples (Fig 6A). A similar phenomenon was also observed when TG2 was extracted from the fascia samples that had been treated with heparinase III in order to degrade the tissue HS (Fig 6A). This suggests that DS/CS (and HS) can mask cryptic sites for TG2 binding in the extracellular space of the human fascia. Thus, to investigate the character of these sites, we repeated our experiment with the TG2 extraction after DS/CS degradation but we also added antibodies against fibronectin and/or collagen type I to the tissue samples taking into account the fact that these molecules are major components of the fascia ECM. The use of chondroitinase ABC treatment combined with the anti-fibronectin antibody enhanced the amount of extracted TG2 insignificantly as compared to the samples that had been extracted only with the serum (Fig 6B). In contrast, the application of the anti-collagen type I antibody together with chondroitinase ABC led to a substantial increase in TG2 extractability from the fascia samples (Fig 6B). Moreover, as was expected, the use of both antisera in combination with chondroitinase ABC did not significantly affect the amount of released TG2 as compared to the samples that had been treated with the enzyme together with the anti-collagen type I antibody (Fig 6B). To summarize, all of these data suggest that DS/CS not only anchors TG2 in the ECM of human tissues but can also regulate the ECM distribution of the enzyme by masking its binding sites, which are especially associated with the collagen network.

### DS/CS can affect TG2 cross-linking activity

**Fig 7:**
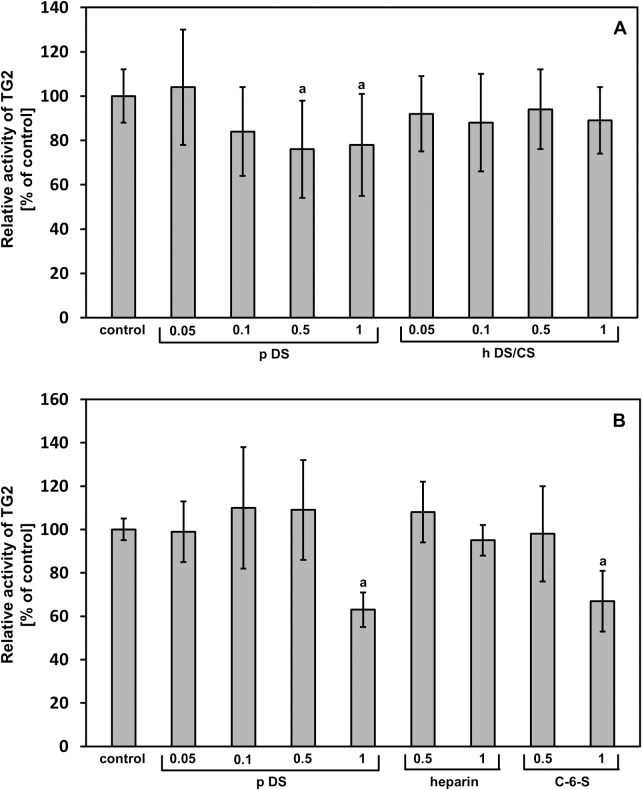
The GAG effect on TG2-dependent transamidating activity. The transamidating activity of the enzyme was estimated as a quantity of biotinylated cadaverine incorporated to CBZ-Gln-Gly (A) or collagen type I (B) without GAGs (control) or with indicated concentrations (μg/ml) of the porcine DS (p DS), the total DS/CS from the fibrotic fascia (h DS/CS), shark cartilage C-6-S or heparin as described under Materials and methods. Relative TG2 activity is expressed as a percentage of the control. The results are means ± S.D. of at least 3 independent experiments. a–differences statistically significant versus control (p ≤ 0.05).

To examine whether DS can influence the TG2-mediated transamidating activity, we studied the enzyme-dependent incorporation of biotinylated cadaverine into an artificial substrate (CBZ-Gln-Gly) in the presence of different concentrations of the porcine intestinal mucosa DS and the total DS/CS from the fibrotic human fascia. As can be seen in Fig 7A, the final effect strongly depends on both the structure and concentration of a GAG. When the porcine DS was applied at the two highest concentrations (1 and 0.5 μg/ml), there was a significant inhibition of TG2 transamidating activity (Fig 7A). However, the other concentrations of this GAG that were used as well as all of the applied concentrations of the human DS/CS failed to show any significant effect on the cross-linking activity of TG2 (Fig 7A). When the porcine DS was used at 1 μg/ml, it also effectively inhibited the TG2-dependent incorporation of cadaverine into collagen type I (Fig 7B). Interestingly, when the shark C-6-S was applied at the same concentration, a similar effect was observed (Fig 7B). In contrast, heparin, which is as strong a binding partner for TG2 [22] as the fibrotic human fascia DS/CS, was similarly unable to affect transamidating activity of the enzyme (Fig 7B).

## Discussion

Our data clearly showed that DS/CS can affect several aspects of TG2 biology that include both the anchoring of the protein in the ECM and the control of its functions in the extracellular space. DS/CS-mediated binding to TG2 has biological relevance as can be observed from our finding that the decorin molecules that are secreted by the human keloid fibroblasts into a culture medium can interact with this protein via their GAG moiety. In addition, our SPR experiment together with the solid-phase binding assay further supports the suggestion that DS is an especially strong binding partner for TG2. However, this binding has some requirements as to the DS structure. Most probably the DS interaction with TG2 involves IdoA residues since our data show that DS has a significantly higher affinity for this protein than CS. However, an appropriate sulfation pattern is also necessary as was observed by the fact that the DSs that differed in respect to this feature manifested a differential binding to TG2. Notably, the porcine DS, which has the highest level of 4,6-O-disulfated disaccharides (the so-called E units) among all of the examined DSs, displayed the lowest affinity for TG2. E units are known to be responsible for DS/CS binding to several growth factors (for example the vascular endothelial growth factor) and receptors [36, 37]. Thus, it is conceivable that other disaccharides such as 2,4-O-disulfated units can contribute to DS/CS binding to TG2. This suggestion is supported by our observation that a positive correlation exists between the content of these disaccharides in the examined DS/CS and the affinity of these GAGs for TG2. Similar to DS/CS, HS also displays a great diversity in respect to its ability to interact with TG2. The HS chains of syndecan-4 bind to this protein while those of syndecan-2 do not [19, 23]. However, what is of great importance is the fact that the fibrotic human fascia DS/CS that strongly bound to TG2 also exerted no inhibitory impact on the transamidating activity of the enzyme. In that case, our observation supports previous finding concerning heparin effect on TG2- mediated cross-linking [22].

Recent studies using site-directed mutagenesis have identified two different sites on the TG2 molecules that are responsible for high affinity heparin binding. The first of these is formed by two clusters of the basic amino acids RRWK (262–265) and KQKRK (598–602), which are localized in the proximity on the TG2 three-dimensional closed structure [34]. In turn, the second heparin binding site is situated within the sequence between the 202 and 222 amino acid residues [23]. These binding sites on TG2 molecules can also be adopted by DS as was observed in our competition experiment, which showed the successful interference of heparin with the interaction between DS and TG2. The occupation of the same basic amino acid clusters by DS and heparin on TG2 molecules may have important biological implications that result from the participation of both of these presumable heparin binding sites to the so-called RGD-independent cell adhesion to the ECM [23, 34]. This phenomenon is mediated via the interactions between the HS chains of the cell-surface situated syndecan-4 and the extracellularly localized TG2 that is complexed with fibronectin [23].

Thus, the use by DS of the same binding site(s) on TG2 molecules as these occupied by HS chains of syndecan-4 can lead to an impairment of cell adhesion in the course of the processes such as fibrosis or scarring that are associated with the ECM accumulation of DS.

*In vivo* DS/CS can also regulate the pool of TG2 that is linked to the ECM thus especially affecting localization of the enzyme. This conclusion results from our observation that the elimination of the fascia DS/CS exposes cryptic sites, which are responsible for TG2 binding, particularly to the collagen network. On the other hand, fibrillar collagens are the substrates for TG2-mediated cross-linking [8]. Then, the finding of a DS-dependent effect on TG2 distribution in the ECM suggests that proportion of DS and collagen may regulate the availability of potential substrates for the enzyme thereby affecting the bio-mechanical properties of tissues. Further confirmation of this hypothesis comes from our finding that some DS/CS that weakly interact with TG2 demonstrated an inhibitory effect on the enzyme-dependent incorporation of cadaverine into both the artificial substrate and collagen type I. The most possible explanation of this phenomenon is the rather indirect action of these GAGs on TG2-mediated cross-linking through their influence on the accessibility of the enzyme substrates. Our unpublished SPR data suggest that Ca^+2^ ions, which are necessary for TG2 to initiate its transamidating activity, strongly impair the ionic interaction between this protein and DS. Thus, in the presence of calcium, negatively charged DS/CS, which have a low affinity for TG2, may be prone to preferential binding to the positively charged substrates of this enzyme. In contrast, under the same conditions, DS with a high affinity for TG2 can still be bound to this enzyme and exert no effect on substrate accessibility. This suggestion results from our observation that GAGs with a high binding potential toward TG2 such as the total DS/CS from fibrotic fascia or heparin did not affect the transamidating activity of the enzyme.

Although collagen type I interacts with DS [38], there is only indirect evidence that this protein can bind to TG2 [39]. Recently, Cardoso et al. [40], using the co-immunofluorescence staining of tissue sections, showed that the staining patterns for TG2 and collagen type I or type III do not overlap. However, TG2 did co-localize with fibronectin and collagen type VI in the ECM [40]. The latter protein also showed ability to directly interact with TG2 in a microplate protein-binding assay [40]. Moreover, collagen type VI, which is ubiquitous in the ECM of tissues, has been found to be intermingled with fibril-forming collagens, mainly collagen type I [41]. In addition, collagen VI is also a binding partner for the core proteins of decorin and biglycan [41]. Thus, cryptic TG2 binding sites localized on collagen network of fascia that were observed in this study after the elimination of the tissue DS may be associated with collagen type VI molecules, which were masked by the DS chains of decorin and biglycan.

Our data suggest that the DS chains of biglycan are especially good binding partners for TG2. Interestingly, both proteins have similar expression patterns because the biosynthesis of both biglycan and TG2 and/or their extracellular manifestations are stimulated after tissue injury, during the progression of fibrosis or arterial wall remodeling [2, 42, 43]. Biglycan is considered to be an element of the damage- and pathogen-associated molecular patterns that mediate their biological effects through toll-like receptors [42]. Notably, the activation of these receptors has also been suggested to be involved in the induction of TG2 transamidating activity after tissue injury [14]. On the other hand, biglycan is found in close association with the fibronectin, elastin and collagen fibrillary networks of tissues. The components of these fibrillary networks are also major substrates for TG2-mediated cross-linking [8, 44, 45]. Thus, the binding of biglycan DS to TG2 that promotes the positioning of the enzyme in crucial ECM sites may play a significant role in the regulation of matrix modification in the course of wound healing, fibrosis, atherosclerosis or small artery remodeling.

## References

[1] Gentile V, Saydak M, Chiocca EA, Akande O, Birckbichler PJ, Lee KN, et al Isolation and characterization of cDNA clones to mouse macrophage and human endothelial cell tissue transglutaminases. J Biol Chem. 1991 1 5;266(1):478–83. 1670766

[2] Wang Z, Griffin M. TG2, a novel extracellular protein with multiple functions. Amino Acids. 2012 2;42(2–3):939–49. 10.1007/s00726-011-1008-x 21818567

[3] Nurminskaya MV, Belkin AM. Cellular functions of tissue transglutaminase. Int Rev Cell Mol Biol. 2012;294:1–97. 10.1016/B978-0-12-394305-7.00001-X 22364871 PMC3746560

[4] Zemskov EA, Mikhailenko I, Hsia RC, Zaritskaya L, Belkin AM. Unconventional secretion of tissue transglutaminase involves phospholipid-dependent delivery into recycling endosomes. Plos One. 2011 4 27;6(4):e19414 10.1371/journal.pone.0019414 21556374 PMC3083433

[5] Chou CY, Streets AJ, Watson PF, Verderio EA, Johnson TS. A crucial sequence for transglutaminase type 2 extracellular trafficking in renal tubular epithelial cells lies in its N-terminal β-sandwich domain. J Biol Chem. 2011 8 5;286(31):27825–35. 10.1074/jbc.M111.226340 21652693 PMC3149372

[6] Balklava Z, Verderio E, Collighan R, Gross S, Adams J, Griffin M. Analysis of tissue transglutaminase function in the migration of Swiss 3T3 fibroblasts: the active-state conformation of the enzyme does not affect cell motility but is important for its secretion. J Biol Chem. 2002 5 10;277(19):16567–75. 10.1074/jbc.M109836200 11867617

[7] Jandu SK, Webb AK, Pak A, Sevinc B, Nyhan D, Belkin AM, et al Nitic oxide regulates tissue transglutaminase localisation and function in the vasculature. Amino Acids. 2013 1;44(1):261–9. 10.1007/s00726-011-1090-0 21984378 PMC3744185

[8] van den Akker J, VanBavel E, van Geel R, Matlung HL, Tuna BG, Janssen GMC, et al The redox state of transglutaminase 2 controls arterial remodelling. Plos One. 2011;6(8):e23067 10.1371/journal.pone.0023067 21901120 PMC3161997

[9] Akimov SS, Krylov D, Fleischman LF, Belkin AM. Tissue transglutaminase is an integrin-binding adhesion coreceptor for fibronectin. J Cell Biol. 2000 2 21;148(4):825–38. 10684262 10.1083/jcb.148.4.825 PMC2169362

[10] Pinkas DM, Strop P, Brunger AT, Khosla C. Transglutaminase 2 undergoes a large conformational change upon activation. PLoS Biol. 2007 12;5(12):e327 10.1371/journal.pbio.0050327 18092889 PMC2140088

[11] Begg GE, Carrington L, Stokes PH, Matthews JM, Wouters MA, Husain A, et al Mechanism of allosteric regulation of transglutaminase 2 by GTP. Proc Natl Acad Sci USA. 2006 12 26;103(52):19683–8. 10.1073/pnas.0609283103 17179049 PMC1750866

[12] Lai TS, Hausladen A, Slaughter TF, Eu JP, Stamler JS, Greenberg CS. Calcium regulates S-nitrosylation, denitrosylation, and activity of tissue transglutaminase. Biochemistry. 2001 4 24;40(16):4904–10. 11305905 10.1021/bi002321t

[13] Stamnaes J, Pinkas DM, Fleckenstein B, Khosla C, Sollid LM. Redox regulation of transglutaminase 2 activity. J Biol Chem. 2010 8 13;285(33):25402–9. 10.1074/jbc.M109.097162 20547769 PMC2919103

[14] Siegel M, Strnad P, Watts RE, Choi K, Jabri B, Omary MB, et al Extracellular transglutaminase 2 is catalytically inactive, but is transiently activated upon tissue injury. Plos One. 2008 3 26;3(3):e1861 10.1371/journal.pone.0001861 18365016 PMC2267210

[15] Huelsz-Prince G, Belkin AM, van Bavel E, Bakker ENTP. Activation of extracellular transglutaminase 2 by mechanical force in the arterial wall. J Vasc Res. 2013;50(5):383–95. 10.1159/000354222 23988702

[16] Johnson TS, El-Koraie AF, Skill NJ, Baddour NM, El Nahas AM, Njloma M, et al Tissue transglutaminase and the progression of human renal scarring. J Am Soc Nephrol. 2003 8;14(8):2052–62. 12874459 10.1097/01.asn.0000079614.63463.dd

[17] Telci D, Collighan RJ, Basaga H, Griffin M. Increased TG2 expression can result in induction of transforming growth factor beta1, causing increased synthesis and deposition of matrix proteins, which can be regulated by nitric oxide. J Biol Chem. 2009 10 23;284(43):29547–58. 10.1074/jbc.M109.041806 19657147 PMC2785588

[18] Verderio E, Gaudry C, Gross S, Smith C, Downes S, Griffin M. Regulation of cell surface tissue transglutaminase: effects on matrix storage of latent transforming growth factor-beta binding protein-1. J Histochem Cytochem. 1999 11;47(11):1417–32. 10544215 10.1177/002215549904701108

[19] Wang Z, Collighan RJ, Gross SR, Danen EH, Orend G, Telci D, et al RGD-independent cell adhesion via a tissue transglutaminase-fibronectin matrix promotes fibronectin fibril deposition and requires syndecan-4/2 α5β1 integrin co-signaling. J Biol Chem. 2010 12 17;285(51):40212–29. 10.1074/jbc.M110.123703 20929862 PMC3001003

[20] Akimov SS, Belkin AM. Cell-surface transglutaminase promotes fibronectin assembly via interaction with the gelatin-binding domain of fibronectin: a role in TGFbeta-dependent matrix deposition. J Cell Sci. 2001 8;114(Pt16):2989–3000. 11686302 10.1242/jcs.114.16.2989

[21] Telci D, Wang Z, Li X, Verderio EAM, Humphries MJ, Baccarini M, et al Fibronectin-tissue transglutaminase matrix rescues RGD-impaired cell adhesion through syndecan-4 and beta1 integrin co-signaling. J Biol Chem. 2008 7 25;283(30):20937–47. 10.1074/jbc.M801763200 18499669 PMC3258940

[22] Scarpellini A, Germack R, Lortat-Jacob H, Muramatsu T, Billett E, Johnson T, et al Heparan sulfate proteoglycans are receptors for the cell-surface trafficking and biological activity of transglutaminase-2. J Biol Chem. 2009 7 3;284(27):18411–23. 10.1074/jbc.M109.012948 19398782 PMC2709370

[23] Wang Z, Collighan RJ, Pytel K, Rathbone DL, Li X, Griffin M. Characterization of heparin-binding site of tissue transglutaminase: its importance in cell surface targeting, matrix deposition, and cell signaling. J Biol Chem. 2012 4 13;287(16):13063–83. 10.1074/jbc.M111.294819 22298777 PMC3339925

[24] Seidler DG, Dreier R. Decorin and its galactosaminoglycan chain: extracellular regulator of cellular function? IUBMB Life. 2008 11;60(11):729–33. 10.1002/iub.115 18800386

[25] Malmstrom A, Bartolini B, Thelin MA, Pacheco B, Maccarana M. Iduronic acid in chondroitin/dermatan sulfate: biosynthesis and biological function. J Histochem Cytochem. 2012 12;60(12):916–25. 10.1369/0022155412459857 22899863 PMC3527884

[26] Koźma EM, Olczyk K, Wisowski G, Głowacki A, Bobiński R. Alterations in the extracellular matrix proteoglycan profile in Dupuytren’s contracture affect the palmar fascia. J Biochem. 2005 4;137(4):463–76. 10.1093/jb/mvi054 15858170

[27] Dolynchuk KN. Inhibition of tissue transglutaminase and epsilon (gamma-glutamyl) lysine cross-linking in human hypertrophic scar. Wound Repair Regen. 1996 Jan-Mar;4(1):16–20. 10.1046/j.1524-475X.1996.40105.x 17129343

[28] Koźma EM, Wisowski G, Olczyk K. Platelet derived growth factor BB is a ligand for dermatan sulfate chain(s) of small matrix proteoglycans from normal and fibrosis affected fascia. Biochemie. 2009 Nov-Dec;91(11–12):1394–404. 10.1016/j.biochi.2009.07.010 19631712

[29] Farndale RW, Buttle DJ, Barrett AJ. Improved quantitation and discrimination of sulphated glycosaminoglycans by use of dimethylmethylene blue. Biochim Biophys Acta. 1986 9 4;883(2):173–7. 3091074 10.1016/0304-4165(86)90306-5

[30] Deakin JA, Lyon M. A simplified and sensitive fluorescent method for disaccharide analysis of both heparin sulfate and chondroitin/dermatan sulfates from biological samples. Glycobiology. 2008 6;18(6):483–91. 10.1093/glycob/cwn028 18378523

[31] Zhao W, McCallum SA, Xiao Z, Zhang F, Linhardt RJ. Binding affinities of vascular endothelial growth factor (VEGF) for heparin-derived oligosaccharides. Biosci Rep. 2012 2;32(1):71–81. 10.1042/BSR20110077 21658003 PMC3190640

[32] Koźma EM, Wisowski G, Latocha M, Kusz D, Olczyk K. Complex influence of dermatan sulphate on breast cancer cells. Exp Biol Med (Maywood). 2014 12;239(12):1575–88. 24912503 10.1177/1535370214538590

[33] Koźma EM, Wisowski G, Kusz D, Olczyk K. The role of decorin and biglycan dermatan sulfate chain(s) in fibrosis affected fascia. Glycobiology. 2011 10;21(10): 1301–16. 10.1093/glycob/cwr065 21543445

[34] Lortat-Jacob H, Burhan I, Scarpellini A, Thomas A, Imberty A, Vives RR, et al Transglutaminase-2 interaction with heparin. Identification of heparin binding site that regulates cell adhesion to fibronectin-transglutaminase-2 matrix. J Biol Chem. 2012 5 25;287(22):18005–17. 10.1074/jbc.M111.337089 22442151 PMC3365763

[35] Belkin AM, Zemskov EA, Akimov SS, Sikora S, Strongin AY. Cell-surface-associated tissue transglutaminase is a target of MMP-2 proteolysis. Biochemistry. 2004 9 21;43(37):11760–9. 10.1021/bi049266z 15362860

[36] Deepa SS, Umehara Y, Higashiyama S, Itoh N, Sugahara K. Specific molecular interactions of oversulfated chondroitin sulfate E with various heparin-binding growth factors. Implications as a physiological binding partner in the brain and other tissues. J Biol Chem. 2002 11 15;277(46):43707–16. 10.1074/jbc.M207105200 12221095

[37] Mikami T, Kitagawa H. Biosynthesis and function of chondroitin sulfate. Biochim Biophys Acta. 2013 10;1830(10):4719–33. 10.1016/j.bbagen.2013.06.006 23774590

[38] Raspanti M, Viola M, Forlino A, Tenni R, Gruppi C, Tira ME. Glycosaminoglycans show a specific periodic interaction with type I collagen fibrils. J Struct Biol. 2008 10:164(1):134–9. 10.1016/j.jsb.2008.07.001 18664384

[39] Juprelle-Soret M, Wattiaux-De Coninck S, Wattiaux R. Subcellular localization of transglutaminase. Effect of collagen. Biochem J. 1988 3 1;250(2):421–7. 2895639 10.1042/bj2500421 PMC1148873

[40] Cardoso I, Stamnaes J, Andersen JT, Melino G, Iversen R, Sollid LM. Transglutaminase 2 interactions with extracellular matrix proteins as probed with celiac disease autoantibodies. FEBS J. 2015 6;282(11):2063–75. 10.1111/febs.13276 25808416

[41] Wiberg C, Hedbom E, Khairullina A, Lamandé SR, Oldberg A, Timpl R, et al Biglycan and decorin bind close to the N-terminal region of the collagen VI triple helix. J Biol Chem. 2001 6 1:276(22):18947–52. 10.1074/jbc.M100625200 11259413

[42] Nastase MV, Young MF, Schaefer L. Biglycan: a multivalent proteoglycan providing structure and signals. J Histochem Cytochem. 2012 12;60(12):963–75. 10.1369/0022155412456380 22821552 PMC3527886

[43] Bakker ENTP, Pistea A, VanBavel E. Transglutaminases in vascular biology: relevance for vascular remodelling and atherosclerosis. J Vasc Res. 2008;45(4):271–8. 10.1159/000113599 18212504

[44] Chen S, Birk DE. The regulatory roles of small leucine-rich proteoglycans in extracellular matrix assembly. FEBS J. 2013 5;280(10):2120–37. 10.1111/febs.12136 23331954 PMC3651807

[45] Schaefer L, Beck KF, Raslik I, Walpen S, Mihalik D, Micegova M, et al Biglycan, a nitric oxide-regulated gene, affects adhesion, growth, and survival of mesangial cells. J Biol Chem. 2003 7 11;278(28):26227–37. 10.1074/jbc.M210574200 12719420

